# Computational Pharmacodynamic Analysis of Cyclopeptides Derived from c[Trp-Phe-D-Pro-Phe] (CJ-15,208), an Unusual Class of Mixed μ/k-Opioid Receptor Ligands Lacking the Traditional Pharmacophores

**DOI:** 10.3390/biomedicines14030580

**Published:** 2026-03-05

**Authors:** Marco Francescato, Hang Liao, Lorenzo Cavina, Andrea Bedini, Luca Gentilucci

**Affiliations:** 1 Department of Chemistry “G. Ciamician”, University of Bologna, Plesso Navile—Ue4, via Gobetti 85, 40129 Bologna, Italy; marco.francescato2@unibo.it (M.F.); hang.liao2@unibo.it (H.L.); lor.cavina@gmail.com (L.C.); 2 Department of Pharmacy and Biotechnology, University of Bologna, 40126 Bologna, Italy; andrea.bedini@unibo.it; 3 Health Sciences & Technologies (HST) CIRI, University of Bologna, Via Tolara di Sopra 41/E, Ozzano Emilia, 40064 Bologna, Italy

**Keywords:** CJ-15,208, opioid receptor, conformational analysis, cyclotetrapeptide, molecular docking, atypical agonist

## Abstract

**Background:** There is currently increasing interest in atypical opioid compounds capable of expanding their clinical applications beyond pain management, including the treatment of psychiatric disorders and substance abuse. In this context, the cyclotetrapeptide c[Trp-Phe-D-Pro-Phe] (CJ-15,208, **1**) and its derivatives represent an unusual class of opioid peptides. This compound was found to be a mixed KOR/MOR antagonist in vitro, but it acted as an agonist in vivo. For its diverse analogues, it appeared that receptors’ affinity, selectivity, and agonist/antagonist activity greatly varied upon modifications to backbone geometry and the 3D display of pharmacophores. **Methods:** We utilized NMR, molecular dynamics, and molecular docking to analyze 3D structures and pharmacodynamic properties of selected representative cyclopeptide analogues of **1**. **Results:** The simulations support that, despite its contradictory functional activity in vitro and in vivo, **1** can bind to the active conformation of receptors in an agonist-like fashion. In general, Trp appeared to be the fundamental pharmacophore in the ligand–receptor complexes. In particular, agonists showed a direct interaction between the indole ring and the carboxylate of the conserved Asp(3:32). **Conclusions:** These studies support a distinctive pharmacodynamic model for this class of compounds, potentially useful for the design of opioid compounds with novel binding/activity profiles and improved therapeutic effects.

## 1. Introduction

Opioid ligands offer therapeutic opportunities that extend well beyond their established role in pain management [1]. While their antinociceptive actions remain central to the treatment of moderate to severe pain, the diverse signaling properties of opioid receptors have opened new avenues in neuropsychiatric research. Modulation of opioid receptors has been implicated in affective regulation, stress responsivity, reward processing, and social bonding, suggesting potential applications in conditions such as depression, anxiety disorders, and substance use disorders [2].

The vast majority of the clinically relevant opioid compounds target the μ-opiod receptor (MOR). Unfortunately, their therapeutic application is limited due to associated risks, such as co-occurring substance use disorders. MOR-selective agonists produce profound antinociception but also algesic tolerance, respiratory depression, and constipation [3]. Drugs that target the δ-opioid receptor (DOR) show promise in pain and mood regulation [4]. However, DOR-selective agonists are generally less potent and can induce seizures. The activation of the nociceptin opioid receptor (NOP) modulates pain, stress, mood, and reward pathways, often producing effects that are anti-opioid or pronociceptive actions in certain contexts [5]. On the other hand, there has been recent interest in molecules that target the κ-opioid receptor (KOR) due to its potential role in developing non-addictive pain therapies and novel treatments for mood and stress-related disorders. Indeed, KOR agonists can produce potent analgesic effects while limiting the harmful gastrointestinal and respiratory side effects typically associated with MOR activation [6]. However, at present, no KOR agonist is used to treat pain in humans, mostly due to relevant side effects, i.e., severe dysphoria, neuropathy-induced astrocyte proliferation and subsequent hyperalgesia, sedation, coordination impairment, and anhedonia. In addition to analgesia, KOR agonists show potential for the treatment of pruritis, multiple sclerosis, Alzheimer’s disease, schizophrenia, immune-mediated diseases such as osteoarthritis, atopic dermatitis, food allergy, gastrointestinal diseases, cancer, and hypoxia and ischemia [7,8]. KOR antagonists are currently debated for their potential antidepressant and anxiolytic effects, as well as for the development of new therapies for addiction, in particular, to cocaine or alcohol [9,10].

Within this frame, recent research has been directed to the identification of new KOR ligands with unusual structures, capable of targeting other ORs simultaneously, with mixed agonist/antagonist opioid functional activity. There is evidence that the ORs do not act in isolation, and their mutual interactions, as well as their interactions with other proteins, may produce clinically useful effects. These multifunctional ligands represent a promising strategy for achieving balanced analgesic efficacy with improved safety and reduced abuse potential, driving significant interest across pharmacological and clinical research fields [11].

In this context, the cyclopeptide c[Trp-Phe-D-Pro-Phe] (CJ-15,208, **1**) emerged as an unusual multi-target opioid ligand (Table 1). This cyclopeptide was isolated from the fermentation broth of a fungus, *Ctenomyces serratus ATCC15502* [12]. CJ-15,208 is distinctive among opioid ligands because it lacks the typical structural pharmacophore, i.e., the protonated amino group commonly required for classical OR binding. Using a guinea pig brain membrane preparation, the in vitro binding affinity of CJ-15,208 was assessed against three ORs [12]. It was found to be a modestly selective mixed KOR/MOR ligand (IC_50_ (nM) KOR 47, MOR 260, and DOR 2600). In the electrically stimulated twitch response assay of rabbit vas deferens, **1** recovered the suppression by a KOR agonist asimadoline (ED_50_ 1.3 μM), indicating that it is an antagonist [12].

*Summary of structure–activity relationship (SAR) studies.* Due to its unusual structure and promising pharmacological profile, **1** attracted some interest, prompting the development of various derivatives, each exhibiting characteristic features (Table 1). The importance of the residues Trp and Phe^4^ was demonstrated by Ala-scan. Indeed, all Ala derivatives suffered a substantial loss in binding affinity, the only exception being c[Trp-Ala-D-Pro-Phe], which maintained high affinity to KOR and MOR [13].

The epimer c[D-Trp-Phe-D-Pro-Phe] ([DTrp]CJ-15,208, **2**) [14] also showed mixed KOR/MOR binding (Table 1) (IC_50_ 3.8, 30, and >1000 nM at KOR, MOR, and DOR, respectively). Interestingly, the replacement of Phe^2^ with Ala gave c[D-Trp-Ala-D-Pro-Phe], which maintained KOR/MOR mixed affinity, while the substitution of Phe^4^ gave a completely inactive peptide.

Subsequent displacement binding tests performed by Aldrich et al. implied that the affinity of **2** for ORs (IC_50_ 30.6, 259, and 2910 nM at KOR, MOR, and DOR) is not superior to that of **1** [15]. The same authors confirmed that L-Trp-containing peptide **1** and stereoisomer **2** (Table 1) did not exhibit any agonist activity at either KOR or MOR in the [^35^S]-GTPγS test.

In addition, both diastereoisomers counteracted the agonist activity of DynA(1–13)NH_2_ [15]. Further, **1** also demonstrated MOR antagonist activity, while **2** showed no antagonist activity at MOR, in contrast to the results reported by Dolle et al. [14].

Unexpectedly, CJ-15,208 (**1**) exhibited robust agonist activity in vivo in the warm-water tail-withdrawal antinociceptive assay, in addition to KOR-selective antagonist activity [13]. The antinociceptive effects of **1** appeared to be mediated by KOR and MOR. Indeed, the effect was suppressed by pretreatment with KOR-selective antagonists (nor-BNI or zyklophin) and by pretreatment with MOR-selective antagonists (β-FNA or CTAP) [13]. The results suggested that **1** acted as a combined KOR/MOR receptor agonist. On the other hand, **2** primarily exhibited KOR antagonism with modest antinociception only at elevated doses [15].

Both **1** and **2** were found to be orally active. Orally administered **2** antagonized centrally administered U50,488-induced antinociception and prevented stress-induced reinstatement of extinguished cocaine-seeking behavior, without affecting locomotor activity [19]. As for **1**, oral administration produced dose-dependent antinociception and KOR antagonist activity and prevented reinstatement of cocaine-seeking behavior [20].

The Ala analogues of **2** were assayed for in vivo antinociception. These analogues displayed pharmacological profiles quite different from the parent peptide. While the analogues exhibited varying OR affinities and KOR/MOR antagonist activity in vitro, they produced potent OR-mediated antinociception in vivo [21]. When D-Trp was replaced by D-Ala, the resulting cyclopeptide exhibited very low KOR affinity.

Subsequently, other SAR studies were conducted on natural **1** and D-Trp isomer **2**. To investigate the role of the indole ring of Trp, De Marco et al. designed the peptides c[(1-MeTrp)-Phe-D-Pro-Phe] and c[D-(1-MeTrp)Phe-D-Pro-Phe] as analogues of **1** and **2,** respectively, in which the indole ring was *N*-methylated. These cyclopeptides showed negligible or very low affinity, highlighting the importance of indoleNH [16]. Also, reversal of stereochemistry at positions 2, 4, or both yielded stereoisomers with different opioid activity profiles and, in general, decreased affinity [22]. In addition, Aldrich et al. explored the effects of substitutions of Trp in **1** and **2**. The substitution altered opioid activity, analgesic potency, metabolic stability, and potential liabilities [23].

Interesting results have been obtained by enlarging macrocycle size. De Marco et al. expanded the macrolactam ring of **1** by insertion of β- or γ-residues. As it turned out, this modification impacted both selectivity and functional activity. Indeed, the 13-membered ring peptide c[Trp-β-Ala-D-Pro-Phe] (**3**, Table 1), containing a β-Ala residue instead of Phe^2^, was a highly selective MOR ligand (Ki 4.1 nM) and was found to be a MOR-selective full agonist [16]. In contrast, the introduction of GABA (*γ*-aminobutyric acid) gave the 14-membered c[Trp-GABA-D-Pro-Phe] (**4**), which showed a lower affinity to MOR but gained a noteworthy DOR affinity (Ki 3.08 nM) and turned out to be a mixed MOR agonist/DOR antagonist (Table 1) [16]. Indeed, the MOR-selective **3** inhibited forskolin-induced cAMP accumulation (IC_50_ 6.1 nM, Emax 90%), suggestive of a full agonist behavior, while the DOR ≫ MOR ligand **4** did not alter forskolin-induced cAMP accumulation in HEK-293 cells expressing human DOR, but it significantly antagonized the effects exerted by DPDPE on forskolin-induced cAMP accumulation (IC_50_ 7.4 nM). Finally, the MOR agonist **3** was shown to elicit potent antinociception in a mouse model of visceral pain upon intraperitoneal administration.

On the other hand, c[D-Trp-Phe-Gly-β-Ala] (**5**, Table 1), a 13-membered analogue of **2** containing β-Ala, revealed high affinity and selectivity to KOR (Ki MOR 1.19 nM, Ki DOR and MOR >10^5^ nM) and G-protein biased agonist activity [17]. Indeed, **5** appeared to be a selective, G-protein-biased KOR agonist that inhibited adenylyl cyclase and activated early-phase ERK1/2 phosphorylation. Peptide **5** neither induced p38MAPK phosphorylation nor increased KOR-dependent, p38MAPK-mediated cell proliferation in astrocytes. Moreover, **5** counteracted U50,488-induced p38MAPK phosphorylation and astrocyte cell proliferation. In vivo, **5** displayed potent antinociception in models of acute nociception, counteracted oxaliplatin-induced thermal hypersensitivity better than U50,488, and was also effective after single or repeated s.c. administration. Administered at a dose that fully alleviated oxaliplatin-induced thermal hypersensitivity, **5** did not alter motor coordination, locomotor and exploratory activities, nor did it induce prodepressant-like behavior [17].

In contrast, the peptide c[D-Trp-Phe-Gly-Ala] (**6**), an analogue of **2** composed of all α-residues, displayed a modest affinity to MOR (Table 1) [18]. Furthermore, introducing GABA at either position 3 or 4 gave the 14-membered c[D-Trp-Phe-GABA-Gly] (7) and c[D-Trp-Phe-Gly-GABA] (**8**), respectively (Table 1). While **7** showed a modest affinity to KOR, **8** was a good and selective MOR ligand [18].

Finally, and quite interestingly, the introduction of two β-Ala residues produced the 14-membered c[D-Trp-Phe-β-Ala-β-Ala] (**9**), the first reported KOR-selective negative allosteric modulator (NAM) (Table 1) [18]. Indeed, in radioligand binding assays, **9** showed a subnanomolar affinity to KOR, albeit it did not completely displace [^3^H]-U69,593 binding, and determined a significant and concentration-dependent decrease in [^3^H]-U69,593 binding to KOR without altering its displacement from the orthosteric site by increasing concentrations of U50,488 or norBNI. Peptide **9** did not inhibit forskolin-induced cAMP accumulation when administered as a single agent to cells expressing heterologous or endogenous h-KOR. However, when **9** was coadministered with the KOR-selective agonist U50,488, it significantly decreased the potency and efficacy of the latter in inhibiting forskolin-induced cAMP accumulation. When **9** was coadministered with norBNI, the concentration-dependent inhibition of cAMP accumulation by U50,488 was significantly shifted further rightward and upward, as compared to the rightward shift induced when only norBNI was coadministered with U50,488. Consistently, **9** decreased U50,488 potency and efficacy in activating ERK1/2 and significantly reduced U50,488 potency and efficacy in inducing astrocytoma cell migration. Accordingly, the potency and efficacy of U50,488 in the presence of norBNI were further decreased by coadministration with **9**, confirming that **9** was a negative allosteric modulator at KOR.

From the SAR studies discussed above, it appears that the two aromatic residues Trp and Phe represent the peptides’ minimal pharmacophoric motif for interacting with the ORs and that modification of structure, ring size, and stereochemistry can tune selectivity to one receptor type over the others. Apparently, the family of CJ-15,208 derivatives can be regarded as a “master set of picklocks”, each capable of “unlocking” one OR subtype or another, plausibly related to the different 3D displays of pharmacophores [24]. Herein, we present a study aimed at investigating the 3D structures of relevant CJ-15,208 derivatives, as well as their pharmacodynamic properties, with the goal of gaining useful information for the future design of molecules with unusual opioid binding and activity profiles. For this purpose, we analyzed the conformations of the peptides by NMR analysis and molecular dynamics and the receptor-bound bioactive conformations by molecular docking simulations [25]. The comparison of in-solution vs receptor-bound conformations allowed for identifying structural determinants and fundamental pharmacophores. The calculated ΔG values of the ligand–receptor complexes correlated well with experimental SAR data, and detailed analysis of the interactions provided insights into the factors underlying agonist or antagonist activity.

## 2. Materials and Methods

*Peptide synthesis and purification* [16,18]. The cyclopeptides were obtained by cyclization of the respective linear precursors under pseudo-high dilution conditions [26]. The linear peptides were prepared by solid-phase peptide synthesis (SPPS) under standard conditions.

In brief, SPPS was performed on Wang resin preloaded with Phe (0.5 g, loading 0.4−0.8 mmol/g), using Fmoc-amino acids (0.6 mmol) and HOBt/TBTU/DIPEA (0.6/0.6/1.2 mmol) as activating agents, in 1:1 DCM/DMF (5 mL). The subsequent Fmoc deprotection steps were performed with 20% piperidine/DMF (5 mL).

The peptides were cleaved from the resin with a TFA/TIPS/water/phenol mixture (15 mL, 7:1:1:1 *v*/*v*). The crude peptides were suspended in ether and collected by centrifugation.

The crude peptides (0.1 mmol) were solubilized in DMF (5 mL) and added over 12 h to a HATU/ DIPEA mixture (0.4/1.0 mmol) in DMF (20 mL) by using a temporized syringe.

The cyclopeptides were isolated by semi-preparative reversed-phase (RP) HPLC using an RP XSelect Peptide CSH C18 OBD column (Waters, Milford, MA, USA) and 4:6 H_2_O/CH_3_CN as the mobile phase. Purities (≥95%) were assessed by analytical RP HPLC, performed with a Phenomenex 3 μm C18 110 A column (Gemini, New York, NY, USA) and mobile phase from 9:1 to 2:8 H_2_O/CH_3_CN. Peptide identity was assessed by ESI MS analysis, performed on a single quadrupole HP 1100 MSD detector (Agilent, Santa Clara, CA, USA).

*NMR analysis.* NMR spectra were recorded on a Bruker BioSpin GmbH (^1^H: 600 MHz, ^13^C: 150 MHz, Billerica, MA, USA) at 298 K in 5 mm tubes, with a peptide concentration of 0.01 M, referenced to the residual nondeuterated DMSO signal as an internal standard (^1^H: 2.50, ^13^C: 39.52 ppm). The assignment of ^1^H NMR resonances was based on 2D gradient-selected COrrelation SpectroscopY (gCOSY) experiments. Variable-temperature (VT) ^1^H NMR experiments were carried out over the range 298–348 K (calibration by the ethylene glycol HO-CH*n* chemical shift separation method). Peptide samples were dissolved in 8:2 DMSO-*d*6/H_2_O; at this concentration, the intramolecular aggregation is usually unimportant. 2D Rotating-frame Overhauser Effect SpectroscopY (ROESY) experiments were done at room temperature in phase-sensitive mode, with a spin-locking field (*γ*b2) of 2000 Hz and a mixing time of 250 ms. Spectra were processed with the hypercomplex approach; peaks were calibrated on the solvent.

*Molecular Dynamics (MD) Simulations.* Only ROESY-derived constraints were included in the restrained MD. Cross-peak intensities were ranked and associated with the distances (Å): very strong, 2.3; strong, 2.6; medium, 3.0; and weak, 5.0. The intensities of the cross-peaks originated from protons separated by known distances (e.g., geminal) were found to match these associations but were discarded. For the absence of H*α*(*i*), H*α* (*i* + 1) ROESY cross-peaks, all of the *ω* bonds were set at 180°. Simulations were conducted with the molecular modeling package Hyperchem 8.0.3 [27] at 300 K and 1 atm by using the AMBER force field [28] in a 30 × 30 × 30 A^3^ box of standard TIP3P models of equilibrated water [29], periodic boundary conditions, dielectric scale factor 1, and a cutoff for the nonbonded interactions of 12 Å. All water molecules closer than 2.3 Å to a solute atom were eliminated, and 50 random structures were generated by a 100 ps simulation at 1200 K; these were subsequently subjected to restrained MD: 50 ps with a 50% scaled force field at 1200 K and then by 50 ps with full distance restraints and a force constant of 7 kcal mol^−1^ A^−2^. Then, the system was cooled in 20 ps to 50 K. Hydrogen-(H-)bond interactions were not included, nor were torsion angle restraints. The resulting structures were minimized by 3000 cycles of steepest descent and 3000 cycles of the conjugated gradient, with convergence = 0.01 kcal A^−1^ mol^−1^. The backbones of the structures were clustered by rmsd analysis.

Unrestrained MD simulations were performed starting with the conformation derived from ROESY in a box of standard TIP3P water for 100 ns at 298 K, using periodic boundary conditions, at constant temperature and pressure (Berendsen scheme, bath relaxation constant of 0.2). For 1–4 scale factors, van der Waals and electrostatic interactions were scaled in AMBER to half their nominal value. The integration time step was set to 0.1 fs. The system coordinates were collected every picosecond.

*Molecular docking.* The ligand molecules were obtained using a systematic conformational search followed by geometry optimization of the lowest-energy structure with MOPAC7 (PM3Method, RMS gradient 0.01) [30]. The simulations were performed with Autodock 4.0 [31]. We used the Lamarckian Genetic Algorithm [32], which combines global search (Genetic Algorithm alone) with local search (Solis and Wets algorithm). Ligands and receptors were further processed using Autodock Tools (ADT) software. Gasteiger PEOE charges [33] were loaded on the ligands in ADT, and solvation parameters were added to the final structure using the Addsol utility of Autodock. Each docking run consisted of an initial population of 100 randomly placed individuals, a maximum number of 200 energy evaluations, a mutation rate of 0.02, a crossover rate of 0.80, and an elitism value of 1. For the local search, the so-called pseudo-Solis and Wets algorithm was applied using a maximum of 250 iterations per local search; 250 independent docking runs were carried out for each ligand. The grid maps representing the system in the actual docking process were calculated with Autogrid. The dimensions of the grids were 100 × 100 × 100, with a spacing of 0.1 Å between the grid points and the center of the cavity left by the ligand after its removal. The simpler intermolecular energy function based on the Weiner force field in Autodock was used to score the docking results. Through a cluster analysis using the tool ClusDOCK from the web server PacDOCK, it was possible to identify a reduced number of clusters for each promising ligand [34]. Results differing by less than 1.0 A in positional root-mean-square deviation (rmsd) were clustered together and were represented by the result with the most favorable free energy of binding. The poses thus obtained were equilibrated by a 5.0 ns of partially restrained MD simulation using the CUDA version of the GROMACS package [35] with a modified version of the AMBER ff03 force field, a variant of the AMBER ff991 potential in which charges and main-chain torsion potentials have been derived based on QM+continuum solvent calculations, and each amino acid is allowed unique main-chain charges. AmberTools [36] was applied to generate Generalized Amber Force Field (GAFF) files for unusual residues. GROMACS molecular topology files (*.gro and *.top) were obtained from the Amber files by Acpype [37]. The MD simulations consisted of 100 ps heating dynamics from 0 to 300 K, followed by equilibration dynamics performed for 5 ns. The MD simulation was performed at constant temperature and volume.

## 3. Results

We investigated the in-solution conformations and the receptor-bound bioactive conformations of the following representative opioid cyclopeptides (see the Introduction): c[Trp-Phe-D-Pro-Phe] (CJ-15,208, **1**) at KOR and MOR; c[D-Trp-Phe-D-Pro-Phe] ([DTrp]CJ-15,208, **2**) at KOR and MOR; c[Trp-βAla-D-Pro-D-Phe] (**3**) at MOR; c[Trp-GABA-D-Pro-Phe] (**4**) at DOR and MOR; c[D-Trp-Phe-Gly-*β*Ala] (**5**) at KOR; c[D-Trp-Phe-Gly-Ala] (**6**) at MOR; c[D-Trp-Phe-GABA-Gly] (**7**) at KOR; c[D-Trp-Phe-Gly-GABA] (**8**) at MOR; and c[D-Trp-Phe-β-Ala-β-Ala] (**9**) as a NAM at KOR/norBNI. These cyclopeptides were prepared under standard conditions, as reported in the literature, by cyclization of the linear precursors obtained, in turn, by SPPS [16,18].

*Conformational analysis in solution.* The conformations of cyclopeptides **1–9** in solution were studied by VT NMR experiments, 2D ROESY analysis, and MD simulations. NMR spectroscopy was performed in 8:2 DMSO-d6/H_2_O, a highly viscous solvent mixture recommended as an excellent representative of the biological environment for the analysis of opioid peptides [38,39]. For most peptides, the spectra showed a single set of resonances, indicating conformational homogeneity or a rapid interconversion between conformers. In contrast, **6** and **7** showed multiple sets of signals, and for these compounds, only the major set was investigated. gCOSY analyses allowed the unambiguous assignment of the resonances.

**Figure 1:**
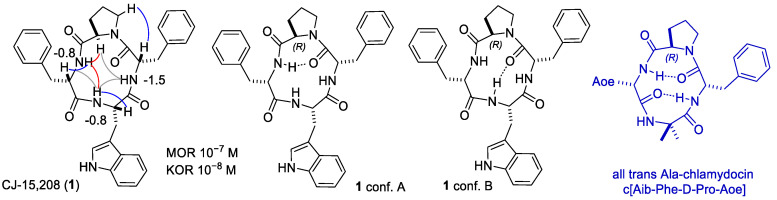
Structure of CJ-15,208 (**1**), with Δδ/Δt parameters for amide protons, as determined by VT-NMR analysis, and selected proton–proton correlations, as determined by ROESY analysis; red, blue, and grey represent strong, medium, and weak correlations, respectively. Conformations A and B of **1**, as determined by unrestrained MD simulations, showing relevant H-bonded secondary structures. For comparison, the all-trans bis-γ-turn conformation of the Pro-containing cyclotetrapeptide chlamydocin (Aib, α-aminoisobutyric acid; Aoe, 2-amino-8-oxo-9,10-epoxy-decanoic acid) is also shown [40].

**Figure 2:**
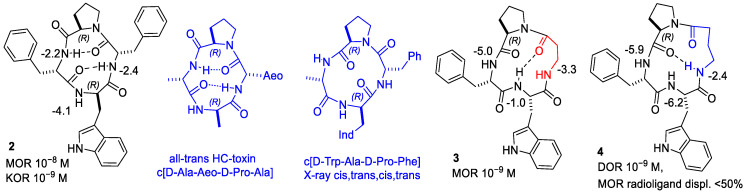
Structures of [D-Trp]CJ-15,208 (**2**), **3,** and **4**, showing Δδ/Δt parameters, as determined by VT-NMR analysis. Relevant H-bonded structures, as deduced by ROESY analysis and molecular dynamics. For comparison, the structures of Pro-containing cyclopeptides that share the same stereochemistry pattern as **2** are also shown, i.e., the all-trans bis-γ-turn conformation of HC-toxin [41], and the X-ray cis,trans,cis,trans conformation of c[D-Trp-Ala-D-Pro-Phe] [14].

**Figure 3:**
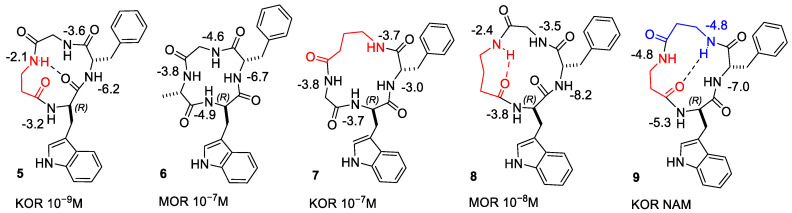
Structures of peptides **5–9**, with Δδ/Δt parameters as determined by VT-NMR analysis. Relevant H-bonded secondary structures have been deduced by ROESY analysis and molecular dynamics.

Variable-temperature (VT) NMR experiments were used to detect if amide protons were plausibly involved in intramolecular H-bonding or were solvent-exposed. Generally, H-bonded amide NH signals display comparatively lower Δδ/ΔT values, |Δδ/ΔT| < 2.0 ppb K^−1^, as compared to solvent-exposed amide. The Δδ/Δt parameters of the cyclopeptides revealed amide protons comparatively less sensitive to increasing temperature, suggestive of involvement in H-bonds (Figure 1, Figure 2 and Figure 3).

Subsequently, the compounds were analyzed by 2D-ROESY in DMSO-*d*6/H_2_O (8:2). The cross peaks between protons were ranked as very strong, strong, medium, and weak, and associated with increasing distances (see Section 2).

*Molecular dynamics.* The estimated distances deduced from ROESY were utilized as restraints for performing simulated annealing and restrained MD simulations [27] in a box of explicit water molecules, using the AMBER force field [28]. The minor conformers of **6** and **7** were not analyzed. In brief, random geometries of each peptide were sampled during a high-temperature unrestrained MD simulation in a box of TIP3P models of equilibrated water molecules. For each random structure, the interproton distances deduced by ROESY were utilized as constraints. The structures were subjected to restrained high-temperature simulation with a scaled force field, followed by a period with full restraints, and then the system was slowly cooled. The resulting structures were minimized with AMBER, and the backbones of the structures were clustered by rmsd analysis.

**Figure 4:**
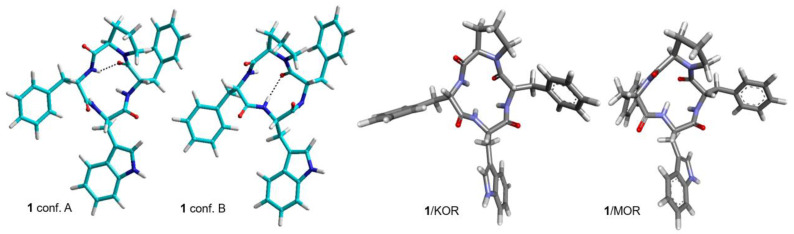
Structures A and B of **1** obtained by NMR and MD simulations (cyan). Structures of **1** bound to KOR and MOR, as predicted by molecular docking (grey).

**Figure 5:**
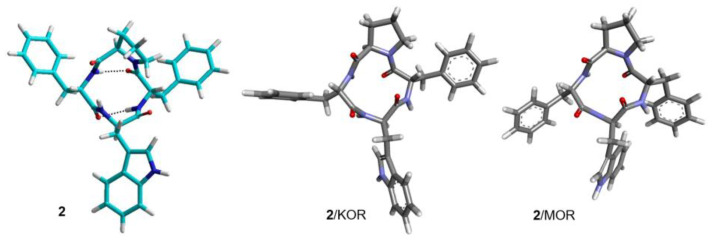
Structure of **2** obtained by ROESY and MD simulations (cyan) [7]. Structures of **2** bound to KOR and MOR, as predicted by molecular docking (grey).

**Figure 6:**
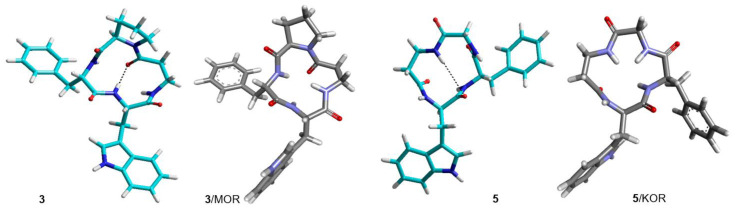
Structures of **3** and **5** obtained by ROESY and MD simulations (cyan). Structures of **3** and **5** bound to MOR and KOR, respectively, as predicted by molecular docking (grey).

**Figure 7:**
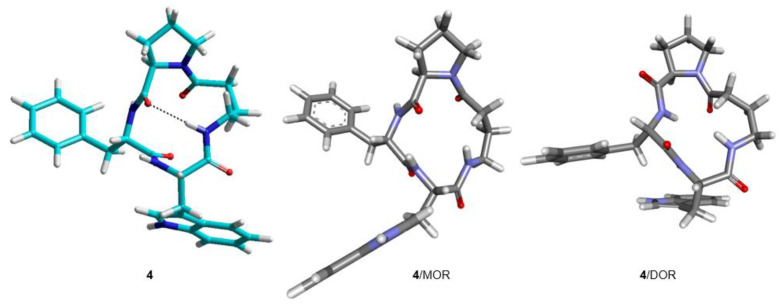
Structure of **4** obtained by ROESY and MD simulations (cyan). Structures of **4** bound to MOR and DOR as predicted by molecular docking (grey).

**Figure 8:**
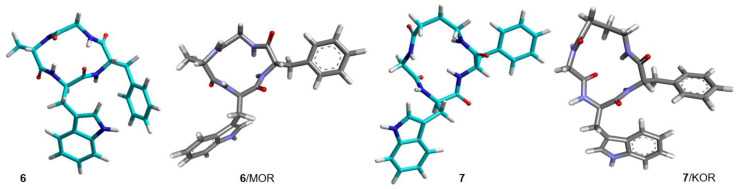
Structures of **6** and **7** obtained by ROESY and MD simulations (cyan). Structures of **6** and **7** bound to MOR and KOR, respectively, as predicted by molecular docking (grey).

**Figure 9:**
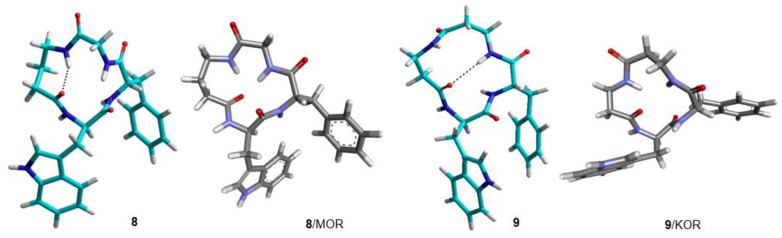
Structures of **8** and **9** obtained by ROESY and MD simulations (cyan). Structures of **8** and **9** bound to MOR and KOR, respectively, as predicted by molecular docking (grey).

The structures with the lowest energy and the least number of restraint violations were inspected. For all cyclopeptides, the procedure gave one major cluster comprising the large majority of the structures; most structures showed explicit H-bonded secondary structures. To investigate the dynamic behavior of the cyclopeptides, the structures were analyzed by unrestrained MD simulations at 298 K in a box of explicit TIP3P equilibrated water molecules. During the simulations, the structures of the backbones were maintained, indicating that these conformations plausibly represented stable minima. Sketches of the structures are shown in Figure 1, Figure 2 and Figure 3, while the 3D structures are shown in Figure 4, Figure 5, Figure 6, Figure 7, Figure 8 and Figure 9.

*Molecular Docking.* Molecular modeling of the prototypic cyclopeptides was performed with Autodock 4.0. using the structures of the receptors deposited in Protein Data Bank: 8f7w (active KOR in complex with dynorphin), 9mql (inactive KOR with nanobodies and isoquinuclidine antagonist), 8f7r (active MOR in complex with endomorphin), 9bjk (inactive MOR bound to a nanobody, naloxone and NAM), 4n6h (inactive DOR bound to naltrindole), and 8vve (inactive KOR with norBNI).

**Figure 10:**
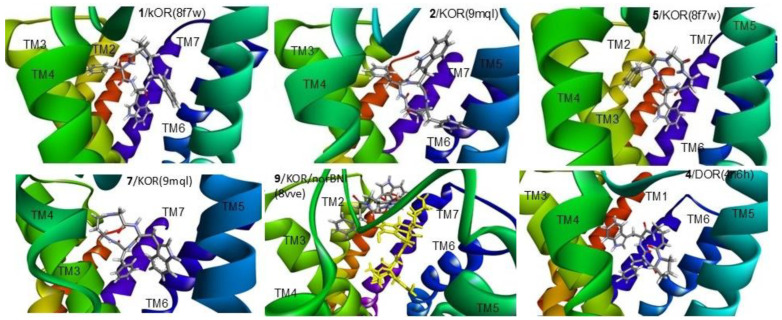
Structures of the complexes predicted for **1, 2, 5, 7** with KOR, for **9** with KOR/norBNI, and for **4** with DOR, as predicted by molecular docking. Ligands are rendered in sticks, and the trans membrane helices are rendered as ribbons. The figure has been elaborated with DSV2025.

**Figure 11:**
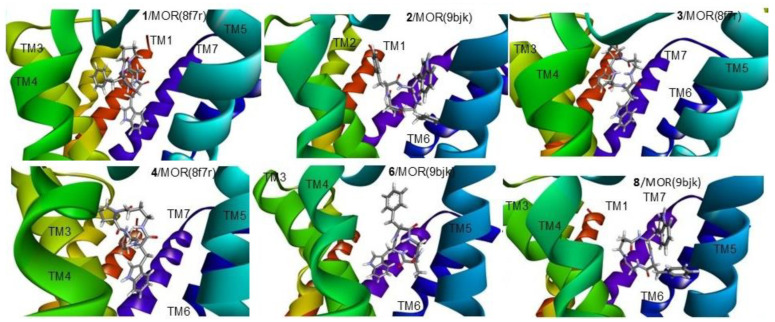
Structures of the complexes predicted for **1**–**4**, **6** and **8** with MOR, as predicted by molecular docking. Ligands are rendered as sticks, and the transmembrane helices are rendered as ribbons. The figure was elaborated with DSV2025.

**Figure 12:**
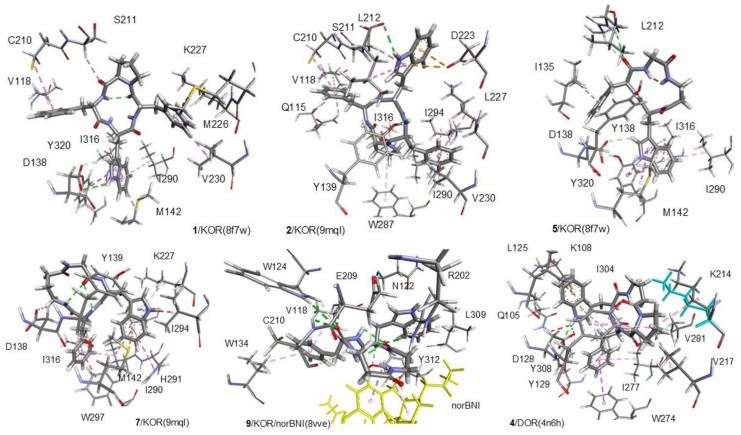
Interactions of **1**, **2**, **6**, **7** with KOR, of **9** with KOR/norBNI, and of **4** with DOR, as predicted by molecular docking. Ligands are rendered as bold sticks, while receptor residues are rendered as thin sticks. The interactions are rendered as dotted lines in the following colors: H-bonds, green; pi–alkyl, grey; pi–pi, pink; and salt bridge, yellow. The figure was elaborated with DSV2025.

**Figure 13:**
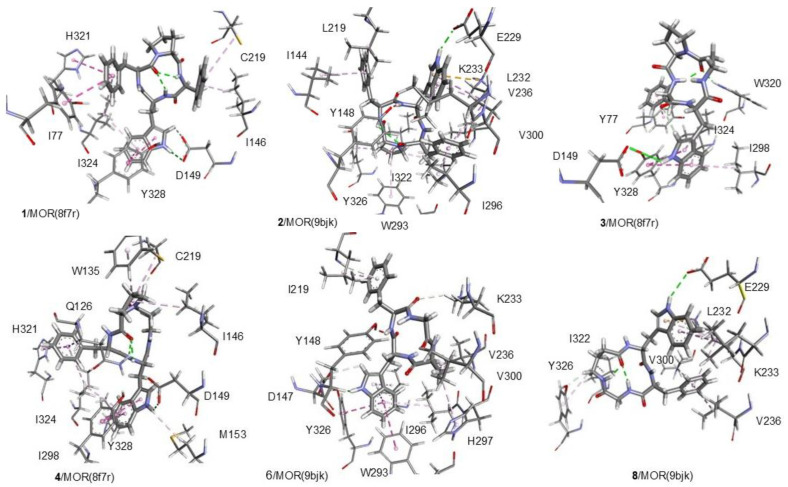
Interactions of **1–4**, **6**, and **8** with MOR, as predicted by molecular docking. Ligands are rendered as bold sticks, while receptor residues are rendered as thin sticks. The interactions are rendered as dotted lines in the following colors: H-bonds, green; pi–alkyl, grey; pi–pi, pink; and salt bridge, yellow. The figure was elaborated with DSV2025.

The best binding poses of the cyclopeptides obtained by the simulations are shown in Figure 10 (for KOR and DOR) and Figure 11 (for MOR). Details of the ligand–receptor interactions are given in Figure 12 (for KOR and DOR) and Figure 13 (for MOR). The interactions were analyzed with BIOVIA DSV2025 [42] and with the PacVIEW tool on the PacDOCK web server [43]. For brevity, herein, only the most relevant features of the complexes are discussed.

In general, the calculated scores were in agreement with the experimental affinity data (ΔG, kcal/mol): **1**/KOR(8f7w) −9.175, **2**/MOR(8f7r) −9.534, **2**/KOR(9mql) −9.211, **2**/MOR(9bjk) −8.655, **3**/MOR(8f7r) −8.327, **4**/DOR(4n6h) −7.810, **4**/MOR(8f7r) −8.530, **5**/KOR(8f7w) −8.048, **6**/MOR(9bjk) −7.655, **7**/KOR(9mql) −7.837, **8**/MOR(9bjk) −6.522, and **9**/KOR/norBNI(8vve) −7.355.

All cyclopeptides but **9** occupy the same location in the crevice delimited by helices (transmembrane, TM) 3, 5, 6, and 6, plus extracellular loops (ELs) 2 and 3. As for the negative allosteric modulator **9**, the best-scoring pose shows the cyclopeptide placed in a spot of KOR/norBNI delimited by TM2, 3, 7, and EL1 and 2. In this pose, the allosteric modulator **9** also makes some contact with the antagonist norBNI (Figure 10 and Figure 12).

## 4. Discussion

The ^1^H NMR analysis of c[Trp-Phe-D-Pro-Phe] (**1**) in DMSO-*d*6/water [16] gave a single set of resonances, suggestive of conformational homogeneity or a rapid equilibrium between conformers. This is consistent with the results obtained in diverse mixtures of solvents, i.e., in acetone-*d*6 [20] and in 1:1 acetone-*d*6/DMSO-*d*6 [12]. The in-solution conformations of c[Trp-Phe^2^-D-Pro-Phe^4^] (CJ-15,208, **1**) have been investigated by NMR analysis in DMSO-*d*6/water and molecular modeling [16] (Figure 1 and Figure 4). The most relevant experimental data of this study are summarized in the following paragraph.

The Δδ/Δt parameters of **1** revealed that the amide protons Phe^4^NH and TrpNH were nearly completely insensitive to increasing temperature, suggesting their plausible participation in strong hydrogen- (H-) bonds (Figure 1) [16]. 2D ROESY yielded a significant number of correlations [16]. However, the ROESY-derived structures analyzed by MD did not show any explicit H-bonds. Nevertheless, clear H-bonds appeared upon inspecting the dynamic behavior of the peptide during unrestrained MD simulations in a water box at RT. The analysis of the trajectories revealed the alternative structures **1**A and **1**B [16]. In structure **1**A, D-Pro appeared embedded into a *γ*-turn centered on D-Pro and stabilized by the explicit H-bond Phe^4^NH-Phe^2^C=O (Figure 1 and Figure 4). Structure **1**B is characterized by an inverse type II β-turn centered on D-Pro-Phe^4^, stabilized by the H-bond TrpNH-Phe^2^C=O. The structures A and B are alternative to those recently calculated by DFT and molecular modeling tools, characterized by the presence of cis peptide bonds [44].

The structures A and B of **1** differ from the all-trans structure of chlamydocin (see next paragraphs) [40] by the reversed display of the peptide bond between Phe^4^-Trp, substantiated by the weak cross-peaks for TrpNH-Phe^4^Hα and the medium cross-peak for TrpNH-TrpHα, and, conversely, the strong cross-peak for TrpNH-Phe^4^NH (Figure 1 and Figure 4).

The in-solution 3D structure of c[Trp-Phe-D-Pro-Phe] ([DTrp]CJ-15,208, **2**) was analyzed as reported for **1**, i.e., by NMR analysis in DMSO-*d*6/water and MD (Figure 2 and Figure 5). The ^1^H NMR showed one set of resonances, as previously reported by Dolle et al. in 1:1 acetone-*d*6/DMSO-*d*6 [14]. Conformational analysis of **2** by 2D ROESY and MD supported the all-trans conformation shown in Figure 2, compatible with the presence of two γ-turns centered on D-Trp and D-Pro (Figure 2 and Figure 5).

The conformations of **1** and **2** are not completely unexpected. The large majority of Pro-containing cyclotetrapeptides adopt an all-transoid amide bond sequence, with or without bis-γ-turn conformation, or a cis,trans,cis,trans amide bond sequence with *i*_i_ symmetry. In general, the peptide bond Xaa-Pro tends to be cis when the preceding amino acid Xaa has the same configuration [45]. Cyclopeptides, all composed of α-residues in L-configuration, are generally conformationally heterogeneous. For instance, NMR experiments of the antibacterial c[Gly-Ser-Pro-Glu] in H_2_O:D_2_O:DMSO-*d*6 (18:1:1) revealed three conformations in a 4:2:1 ratio. The major conformer adopted a reverse-turn conformation and had two cisoid amide bonds at Ser-Pro and Glu-Gly [46].

The inversion of the configuration at any residue of the sequence and/or the nature of the side chains may reduce conformational chaos. The in-solution conformations of c[Gly-Phe-D-Pro-Ala] and c[Pro-D-Phe-Pro-D-Phe] have been studied by NMR in CDCl_3_/DMSO-*d*6 mixtures and by circular dichroism spectroscopy in diverse solvents. These studies indicate that conformations are solvent-dependent [47]. The conformation of c[Gly-Phe-D-Pro-Ala] in CDC1_3_ is characterized by four transoid amide bonds and at least one γ-turn 3-1 intramolecular H-bond. The conformation of c[Pro-D-Phe-Pro-D-Phe] in CDCI_3_ has four transoid amide bonds and two inverse γ-turns. As the mole fraction of DMSO in chloroform increases, cis Xaa-Pro amide bond conformations are found. In neat DMSO-*d*6, c[Pro-D-Phe-Pro-D-Phe] has only cis D-Phe-Pro amide bonds [48].

HC-toxin, a metabolite of *Helminthosporium carbonum*, c[D-Ala-Aeo-D-Pro-Ala], was analyzed by NMR in chloroform [41,49]. In this solvent, the compound gave a single set of resonances, and the results of NOESY analysis were consistent with the all-trans bis-γ-turn conformation (Figure 2).

Other compounds with slightly diverse structures gave different results. Kato et al. used NMR spectroscopy to analyze the conformations of c[D-Tyr(Me)-Ile-Pro-Leu] and c[D-Tyr(Me)-Ile-Pro-D-Leu]. The conformation of the first was cis,trans,trans,trans and that of the second was cis,trans,cis,trans [50]. The 3D structures of chlamydocin, c[Aib-Phe-D-Pro-Aoe] (Aoe, 2-amino-8-oxo-9,10-epoxy-decanoic acid; Aib, α-aminoisobutyric acid), and c[Aib-Phe-D-Pro-Ala] were investigated by NMR in DMSO-*d*6. Two conformations of these molecules were present in a 6:4 ratio, one all-trans was characterized by two γ-turns, (Figure 1), and the other cis,trans,trans,trans was characterized by a β-turn structure centered on Aoe-Aib [40]. On the other hand, chlamydocin-hydroxamic acid derivatives, in which Aib was substituted by L- or D-configured Ala, i.e., c[Ala-Phe-D-Pro-Asu(NHOH)] or c[D-Ala-Phe-D-Pro-Asu(NHOH)] (Asu, amino suberic acid), showed only bis-γ-turn conformation in chloroform and also in DMSO, indicating that the presence of Aib is the reason for the two different conformations [51].

Dolle et al. discussed the 3D structure of c[D-Trp-Ala-D-Pro-Phe], a cyclopeptide derived from **2** by substitution of Phe^2^ with Ala. Based on computational analysis, the authors proposed the all-trans conformation. However, an X-ray analysis showed that c[D-Trp-Ala-D-Pro-Phe] crystallized in the cis,trans,cis,trans conformation (Figure 2), with no intramolecular H-bonds [14].

More recently, the in-solution conformation of the chlamydocin analogue c[Ala-Phe-D-Pro-Aoe] was investigated by X-ray diffraction and NMR spectroscopy. NMR spectra in CDCl_3_ were composed of two sets of similar resonances in a ∼1:1 ratio. The Phe–Pro amide bond bore a trans configuration in one steroisomer, which converted to a cis configuration, giving the second steroisomer [52].

From the examples discussed above, it appears that peptides composed of all α-amino acids can display multiple conformations in equilibrium, albeit sharing the same stereochemistry patterns [53]. On the other hand, the introduction of β- or γ-residues is expected to render the structures easier to synthesize and conformationally more stable [54,55]. In addition, despite the extra C-C bonds with respect to α-amino acids, the introduction of β- or γ-amino acidic building blocks can promote well-defined structures stabilized by H-bonds.

From this perspective, the 13-membered cyclopeptides **3** and **5** and the 14-membered **4**, **7–9** have been designed as conformationally alternative analogues of **1** and **2**. Figure 3, Figure 6, Figure 7, Figure 8 and Figure 9 show the conformations of 13- and 14-membered cyclopeptides, as determined by NMR analysis and molecular dynamics, most of which present explicit H-bonds.

The receptor-bound structures of **1–9**, as determined by molecular docking analysis, are shown in Figure 10, Figure 11, Figure 12 and Figure 13. The structure of the complex **1**/KOR (active conformation) shows the peptide deeply inserted within the helices TM3, 5, 6, 7 (Figure 10 and Figure 12). The sequence of the peptide can be read in a counterclockwise direction. Several interactions contribute to stabilizing the complex. The carboxylate side chain of Asp138 (3:32 in the Ballesteros–Weinstein numbering scheme) shows a bifurcated interaction with the ligand’s Trp indole, i.e., a conventional H-bond (2.95 Å) with IndNH, plus a carbon–hydrogen (C-H) H-bond with IndH2. Other relevant interactions are: IndAr-Y320 (pi–pi stacking), IndAr-I316 (pi–alkyl), IndAr-I290 (pi–alkyl), IndAr-M142, Ph^2^-V230 (pi–alkyl), Ph^2^-M226 (Pi–S), Ph^2^-K227C-chain (pi–alkyl), Ph^4^-C210 (pi–alkyl), Ph^4^-V118 (pi–alkyl), and ProC=O-S211Hα (C-H H-bond).

The structure of the complex **1**/MOR (active conformation) differs in several aspects. The sequence (Figure 11 and Figure 13) can be read in a clockwise direction. Nevertheless, this complex also shows the bifurcated interaction between Asp149(3:32) and IndNH, i.e., the H-bond between AspCOO^−^ and IndNH, and the C-H H-bond with IndH2. Other stabilizing interactions are: IndAr-Y328, Ph^2^-i324, Ph^2^-I77,Ph^2^-H321, Ph^4^-C219, and Ph^4^-I146.

In contrast to **1**/KOR and **1**/MOR, the structures of **2**/KOR (Figure 10 and Figure 12) and **2**/MOR (Figure 11 and Figure 13) (inactive conformations) do not show any contacts between TrpInd and Asp(3:32). The cyclopeptides appear still inserted within TM3, 5, 6, and 7, but the ring is positioned upside-down, with the indole of Trp pointing outwards. Yet, the complexes are stabilized by a number of interactions, accounting for the good scores.

Cyclopeptide **3** is a potent and selective MOR agonist. In the complex **3**/MOR(active), the peptide sequence can be read in a clockwise direction, showing distinctive interactions involving the indole ring of Trp: IndNH-Asp149(3:32) (H-bond), IndAr-Y328, IndAr-I298, IndAr-I324, Ph–Y77, and βAlaC=O-W320IndNH (Figure 11 and Figure 13).

As for the mixed MOR agonist/DOR antagonist **4**, the complex **4**/MOR(active) shows the macrolactam ring in an anticlockwise direction, and the main interactions are: IndNH-Asp149(3:32), IndAr-Y328, IndAr-I324, IndAr-I298, IndAr-M153, Ph-I324, Ph-H321, Ph-N126C-chain, Pro-W135, Pro-C219, Pro-I146, and PheC=O-N126CONH_2_ (Figure 11 and Figure 13). The complex **4**/DOR(inactive) is characterized by the peptide’s clockwise direction. Trp indoleNH still shows the H-bond with Asp128(3:32) and also an H-bond with Y308OH carboxylate, but the orientation of the indole ring is quite different (Figure 10 and Figure 12).

The molecular docking of the KOR-selective agonist **5** accounted for its ability to bind to the active conformation of the receptor. The complex **5**/KOR(active) presents a clockwise macrocycle, with the bifurcated interaction between Asp138(3:32)carboxylate and the indole ring: IndNH-Asp138COO- (H-bond) and IndH2-Asp138COO- (C-H H-bond). Other interactions involving the indole ring are: IndAr-Y320, IndAr-M142, and IndAr-I290.

The complex **6**/MOR(inactive) shows Trp-indole pointing towards Asp147(3.32)carboxylate, without forming any H-bond interactions with IndNH (Figure 11 and Figure 13). The stabilizing contacts about the indole ring are: IndH2-AspCOO- (C-H H-bond), IndNH-Y326OH (H-bond), IndAr-Y236 (pi–pi stacking), and IndAr-W293 (pi–pi stacking).

Cyclopeptide **7** is a modest ligand of KOR. The calculated complex **7**/KOR(inactive) shows that Trp is situated opposite to Asp138(3:32), making contacts with I294, H291, M142, and K227 (Figure 10 and Figure 12).

Similar to the complex **2**/MOR(inactive), the complex **8**/MOR(inactive) is characterized by the outwards position of the indole ring. For both peptides, the macrocycle can be read in a clockwise direction, with IndNH H-bonded to E229COO^−^ (Figure 11 and Figure 13).

Finally, the simulation for KOR-selective NAM **9** was performed with the complex KOR(inactive)/norBNI (pdb: 8vve). In the resulting complex **9**/KOR/norBNI, **9** is adjacent to the antagonist norBNI, within the top of TM2, 3, 7, and the extracellular loops EL1 and 2. The interactions with the receptor are: TrpIndH2-N122C=O (C-H H-bond), TrpHα–Y312C=O (C-H H-bond), IndAr-L309 (pi–alkyl), PheAr-V118 (pi–alkyl), PheAr-V134 (pi–alkyl), PheC=O–W124IndH2 (C-H H-bond), PheC=O–W124IndNH (H-bond), PheC=O–C210NH (H-bond), PheC=O–E209Hα (C-H H-bond), βAla^3^–C210C=O (C-H H-bond), βAla^3^Hβ–E209COO- (C-H H-bond), and βAla^4^C=O-R202guanidine (H-bond). In addition, there are also interactions between **9** and norBNI: βAla^3^–norBNIAr (pi–alkyl) and βAla^3^–norBNIchain (alkyl–alkyl). These interactions seem coherent with the efficacy of **9** to bind the receptor at an allosteric site, stabilizing the antagonist-bound inactive conformation.

For comparison, the docking of **9** was also performed with the inactive KOR receptor (pdb: 8vve) in the absence of norBNI. The simulations showed that **9** can actually bind to the receptor at the orthosteric site with appreciable ΔG (−7.355 kcal/mol), only slightly higher with respect to the ΔG calculated for **9**/KOR/norBNI.

From the inspection of the receptor-bound structures predicted for **1–9**, it can be deduced that, despite the well-known conformational heterogeneity of cyclotetrapeptides, the compounds tend to maintain at the receptor 3D all-trans structures very similar to those adopted in solution. For a comparison of the 3D geometries determined by NMR analysis/MD and the bioactive conformations of the compounds docked in the diverse receptors, see Figure 4, Figure 5, Figure 6, Figure 7, Figure 8 and Figure 9.

In addition, it can be perceived that agonist binding poses display direct interactions between the indole ring of Trp and the carboxylate side chain of the conserved Asp(3:32). Conversely, antagonists, in general, appear to adopt diverse poses at the receptors, not showing clear indoleNH-Asp interactions. This unusual Ind-Asp interaction has been reported in the literature a few times in the opioid peptide field [56,57,58,59,60]. Interestingly, it has been shown that the presence of substituents at the indole of D-Trp-containing opioid peptides can tune receptor affinity and enzymatic stability, allowing a measurable effect on central antinociception in mice after intraperitoneal administration [61]. The large majority of opioid agonists are known to bind to the respective receptors by a salt bridge between the protonated amine of the ligand and the conserved Asp(3:32) in the Ballesteros–Weinstein numbering scheme (see Section 1). Plausibly, the Ind-Asp(3:32) interaction acts as a partial surrogate for the salt bridge with the receptor [62].

## 5. Conclusions

In this work, we investigated the pharmacodynamics of an atypical class of OR ligands, i.e., cyclotetrapeptides derived from CJ-15,208 (**1**). SAR studies showed that modifications of the cyclopeptide sequence, in particular, inversion of stereochemistry and/or exchange of α-residues with β- or γ-residues, clearly tune receptor affinity, selectivity, and functional activity. It is generally accepted that variations in the biological activities of cyclic peptides can be rationalized in conformational terms [63]. Unfortunately, cyclic tetrapeptides often fail to adopt well-defined and stable conformations, making it challenging to correlate the spatial arrangement of their pharmacophores with experimentally determined bioactivity.

From this perspective, we performed structural analysis of representative selected peptides in solution, by NMR and molecular dynamics, and we compared the resulting 3D geometries with the receptor-bound bioactive structures simulated by molecular docking. It can be inferred that the backbone structures of the isolated cyclopeptides are largely preserved upon binding, remaining essentially unchanged within the receptor binding sites. Simulations support that, despite its contradictory functional activity observed in vitro and in vivo [12,15], **1** seems capable of binding to the active conformation of the receptors in an agonist-like fashion. This plausibly suggests that the lack of observed agonist effects may depend on the experimental readouts used (twitch response assay, [^35^S]-GTPγS assay), while the compound may, in fact, be able to activate other signaling pathways as an agonist.

Intriguingly, agonist peptides have a certain attitude for placing the indole ring of Trp against the carboxylate side chain of the conserved Asp(3:32). When a direct H-bond forms between Asp(3:32) and indoleNH, it appears to partially compensate for the absence of the classical salt bridge with the ligand’s protonated amino group, thereby contributing to stabilization of the receptor’s active conformation. This result is not entirely surprising: salvinorin A, a naturally occurring psychoactive terpene from *Salvia divinorum*, neither contains a nitrogen atom nor forms ionic interactions with the receptor, yet it is a highly selective and potent KOR agonist [64,65].

The distinctive pharmacodynamic model emerging from these studies may support the development of novel opioid ligands with atypical structures and promising therapeutic potential, particularly as non-addictive analgesics, treatments for psychiatric disorders, or agents for the management of substance use disorders such as alcohol or cocaine abuse.

## Figures and Tables

**Table 1:** Sequences and structures of cyclopeptides **1–9** (Ind, 3′-indolyl), along with their OR preferences and literature references. Ring size from 12 to 14 is also indicated.

Compd.	Sequence	Structure	Rec. Affinity (nM)/Activity	Refs.
**1** (CJ-15,208)	c[Trp-Phe-D-Pro-Phe]	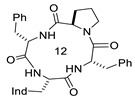	IC_50_ KOR 47; IC_50_ MOR 260/antagonist at MOR and KOR In vitro antagonist/in vivo agonist at MOR and KOR	[12,13]
**2** ([DTrp]CJ-15,208)	c[D-Trp-Phe-D-Pro-Phe]	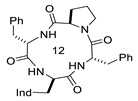	IC_50_ KOR 3.8; IC_50_ MOR 30/antagonist at MOR and KOR IC_50_ KOR 30.6; IC_50_ MOR 259/antagonist at KOR; non-agonist at MOR	[14,15]
**3**	c[Trp-β-Ala-D-Pro-Phe]	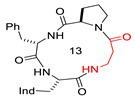	Ki MOR 4.1/agonist	[16]
**4**	c[Trp-GABA-D-Pro-Phe]	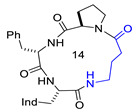	Ki DOR 3.08; Ki MOR 88.3 (radioligand displacement <50%)/MOR agonist; DOR antagonist	[16]
**5**	c[D-Trp-Phe-Gly-β-Ala]	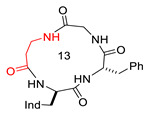	Ki KOR 1.19/G protein biased KOR agonist	[17]
**6**	c[D-Trp-Phe-Gly-Ala]	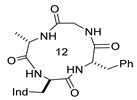	Ki MOR 111.2/non-agonist	[18]
**7**	c[D-Trp-Phe-GABA-Gly]	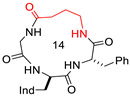	Ki KOR 102/partial agonist	[18]
**8**	c[D-Trp-Phe-Gly-GABA]	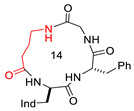	Ki MOR 35.27 non-agonist	[18]
**9**	c[D-Trp-Phe-β-Ala-β-Ala]	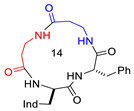	Ki KOR 0.55 (radioligand displacement ≪50%)/NAM	[18]

## Data Availability

The original contributions presented in this study are included in the article. Further inquiries can be directed to the corresponding author.

## Abbreviations

The following abbreviations are used in this manuscript:

OR	opioid receptor
MOR	μ-opioid receptor
DOR	δ-opioid receptor
KOR	κ-opioid receptor
NAM	negative allosteric modulator
SAR	structure-activity relationship
Ind	3′-indolyl
RP HPLC	reversed-phase high-performance liquid chromatography
Aoe	2-amino-8-oxo-9,10-epoxy-decanoic acid
Aib	α-aminoisobutyric acid
H-	hydrogen-
C-H	carbon–hydrogen
VT	Variable temperature
gCOSY	gradient-selected COrrelation SpectroscopY
ROESY	Rotating-frame Overhauser Effect SpectroscopY
SAR	structure–activity study
MD	molecular dynamics
TM	transmembrane
EL	extracellular loop
